# Association of body temperature with obesity. The CoLaus study

**DOI:** 10.1038/s41366-018-0218-7

**Published:** 2018-09-24

**Authors:** François Bastardot, Pedro Marques-Vidal, Peter Vollenweider

**Affiliations:** Department of Medicine, Internal Medicine, Lausanne University hospital, Lausanne, Switzerland

**Keywords:** Epidemiology, Signs and symptoms

## Abstract

**Background and aims:**

There is conflicting evidence regarding the association between body temperature and obesity. We aimed to assess the associations between body temperature and several adiposity and metabolic markers according to gender and menopausal status in a large population-based sample.

**Methods:**

The data collected between 2009 and 2012 from 4224 participants (mean age 57.3 ± 10.4 years, 2225 women) of the CoLaus study (Lausanne, Switzerland). Body temperature was measured at the tympanic membrane.

**Results:**

Mean body temperature was 36.1 ± 0.4, 36.4 ± 0.4 and 36.3 ± 0.4 °C in men, premenopausal, and postmenopausal women, respectively (*p* < 0.001). In men and postmenopausal women, body temperature was positively and significantly (*p* < 0.05) associated with body mass index (Spearman correlation coefficients 0.157 and 0.083, respectively), waist (*r* = 0.163 and *r* = 0.104), waist to hip ratio (*r* = 0.187 and *r* = 0.132), body area (*r* = 0.094 and *r* = 0.085), resting heart rate (*r* = 0.227 and *r* = 182), glucose (*r* = 0.104 and *r* = 0.088) and insulin (*r* = 0.148 and *r* = 0.117). Except for body area and BMI in postmenopausal women, all associations remained significant after multivariable adjustment. In premenopausal women, body temperature was positively associated with resting heart rate (*r* = 0.140) and insulin (*r* = 0.170), and no significant associations were found after multivariable adjustment.

**Conclusion:**

Body temperature is strongly associated with obesity markers in men and postmenopausal women. The absence of association in premenopausal women might be due to the influence of the menstrual cycle.

## Introduction

Body temperature has been used to assess and monitor disease since the Greek Antiquity [1]. Body temperature is controlled by the thermoregulatory center located in the anterior hypothalamus and results from the complex balance between metabolic processes, muscle activity, and possibly the microbiome [2]. Body temperature is also influenced by the external environment via radiation or conduction [3]. For adequate body functioning, body temperature has to be kept constant within a narrow range, at the cost of a significant metabolic expense [4, 5]. Body temperature varies according to physiological (gender, age, and menstrual cycle) [6] and pathological (infection, inflammation, and neoplasia) [7] conditions, and can also be modulated by the consumption of drugs, such as paracetamol, non-steroidal anti-inflammatory drugs (NSAIDs), or corticosteroids.

Among the physiological determinants of body temperature, body mass index (BMI) have been shown to be positively associated with body temperature in some studies [8–10] but not in others [11–13]. A better insulation due to a thicker layer of subcutaneous adipose tissue could explain the higher temperature among the obese [14, 15]. Importantly, most studies were conducted using small sample sizes (42 women and 18 men for [11]) or using devices such as swallow able pill-size sensors, which are not easily applicable in large samples [9, 13]. Indeed, with the exception of a large American study [10] and a Swedish study conducted in the eighties among 816 men [8], no study assessed the association between body temperature and obesity or metabolic markers in the general population.

We thus aimed to assess the associations between body temperature and adiposity and metabolic markers according to gender and menopausal status in a large population-based sample.

## Methodology

### Study design

The CoLaus Study (www.colaus-psycolaus.ch) is a prospective study designed to assess the prevalence of cardiovascular risk factors and to identify new molecular determinants of cardiovascular disease in the population from Lausanne (Switzerland). The baseline and the follow-up methodologies of the CoLaus study have been reported previously [16, 17]. Briefly, recruitment began in June 2003 and ended in May 2006. The follow-up visit was performed between April 2009 and September 2012 and was similar to the baseline evaluation. As body temperature was collected only at the follow-up visit, only data from this visit were used.

### Anthropometric data

Participants were asked to attend the outpatient clinic at the Lausanne university hospital in the morning after an overnight fast. The data were collected by trained field interviewers in a single visit lasting about 60 min. Participants had to be fasting, take their medication as usual, avoid strenuous physical activity during the previous 12 h and abstain from consuming caffeine or alcohol-containing beverages during 24 h before the analysis.

Body weight and height were measured with participants standing without shoes in light indoor clothing. Body weight was measured in kilograms to the nearest 0.1 kg using a Seca™ scale (Seca, Hamburg, Germany). Height was measured to the nearest 5 mm using a Seca™ height gauge (Seca, Hamburg, Germany). BMI was defined as weight (kg)/height^2^ (m^2^). Underweight was defined as BMI <18.5 kg/m^2^; normal weight as BMI ≥18.5 and <25 kg/m^2^; overweight as BMI ≥25 and <30 kg/m^2^ and obesity as BMI ≥30 kg/m^2^.

Waist circumference was measured twice with a non-stretchable tape over the unclothed abdomen at the mid-point between the lowest rib and the iliac crest. Hip circumference was also measured twice at the greater trochanters. For waist and hip, the mean of the two measurements was used and waist-to-hip ratio (WHR) was calculated.

Fat mass was assessed by electrical bioimpedance in the lying position after a 5-min rest using the Bodystat^®^ 1500 body mass analyzer (Bodystat Ltd, Isle of Man, England). This device has been shown to correlate well (*r* = 0.968) with measurements from dual energy X-ray absorptiometry (DEXA) [18]. In a subset of 794 CoLaus women who had also their body composition assessed by DEXA, the correlation between fat mass estimated by bioimpedance and DEXA was 0.852 (*p* < 0.001). All metallic adornments were removed, and measurement was performed after a 5-min rest in the lying position. The electrodes were positioned in the right side of the body according to the manufacturer’s instructions. Care was taken to ensure that the participants did not touch any metallic component of the bed and that the inner part of the thighs did not touch each other. Results were obtained as percentage (%BF); body fat mass was calculated as weight × %BF and expressed in kg. Non-fat mass was obtained by subtracting body fat mass from body weight. (Non) fat mass indexes were calculated as (non) body fat mass (kg)/height^2^ (m^2^). Body area was assessed using the method of Mosteller [19].

### Temperature measurement

Body temperature was measured in degrees Celsius (°C) to the nearest 0.1 °C using a tympanic thermometer (Genius™ 2, Covidien, Dublin, Ireland) according to the manufacturer’s instructions. The measurement was performed in a temperature-controlled room ~20 min after the participant’s admittance.

### Other data

Smoking status was categorized into never, former, and current smoker. Menopause was defined as the absence of menstruations for >1 year. All drugs (prescribed or over the counter) were systematically screened for acetaminophen, NSAIDs or corticosteroids. Resting heart rate was measured thrice on the right arm, after at least 10 min rest in the seated position, using an Omron^®^ HEM-907 automated oscillometric sphygmomanometer (Matsusaka, Japan). Values averaged between the last two readings were used.

Most biological assays were performed by the clinical laboratory of the Lausanne university hospital on fresh blood samples within 2 h of blood collection. The following analytical procedures (with maximum inter and intra-batch CVs) were used on cobas^®^ 8000, Roche Diagnostics, Basel, Switzerland: glucose by hexokinase (1.6%; 0.8%); high sensitive CRP by immunoturbidimetry HS (8.0%; 7.4%); insulin by ECLIA (electrochemiluminescence method) (3.7%; 1.5%). Care was taken that no hemolysis was present so not to bias the results. The assay has been validated and is used for diagnostic procedures, and the technical documentation can be obtained from the authors upon request.

### Inclusion and exclusion criteria

Participants were excluded if they (1) missed data for temperature; (2) missed data for BMI, waist and hip; (3) reported regular or occasional use of acetaminophen, NSAIDs or corticosteroids; (4) presented with an inflammatory syndrome, defined as a high-sensitivity C-reactive protein (hs-CRP) level ≥20 mg/l, and (5) missed data regarding menopausal status (women only). For sensitivity analyses, participants were further excluded if they missed data for bioimpedance.

### Statistical analysis

Statistical analyses were performed with Stata^®^ version 14.1 (Stata Corporation, College Station, TX, USA). As body composition and adiposity markers differ considerably by gender, analyses were stratified by gender. As menstrual cycle influences body temperature in women, a further stratification on menopausal status was performed. Due to their distribution, hs-CRP and insulin were log transformed prior to analyses. Results were expressed as mean ± standard deviation for continuous data or number of participants (percentage) for categorical data. Bivariate analyses were performed using Student’s *t*-test or analysis of variance for continuous data and *χ*^2^-test for categorical data. Bivariate associations between temperature and adiposity and metabolic markers were assessed by Spearman correlation. Multivariable associations between body temperature and continuous markers were assessed using linear regression and the results were expressed as standardized coefficients, which can be interpreted as multivariable-adjusted correlation coefficients. Multivariable associations between body temperature and BMI categories were assessed using analysis of variance and results were expressed as multivariable-adjusted mean ± standard error; test for a linear trend was performed using command **contrast p**. of Stata^®^. Statistical significance was considered for a two-sided test with *p* < 0.05.

### Ethical statement

The institutional Ethics Committee of the University of Lausanne, which afterwards became the Ethics Commission of Canton Vaud (www.cer-vd.ch) approved the baseline CoLaus study (reference 16/03, decisions of 13th January and 10th February 2003); the approval was renewed for the first follow-up (reference 33/09, decision of 23rd February 2009). The full decisions of the CER-VD can be obtained from the authors upon request. The study was performed in agreement with the Helsinki declaration and its former amendments, and in accordance with the applicable Swiss legislation. All participants gave their signed informed consent before entering the study.

## Results

### Sample selection and characteristics

**Fig. 1:**
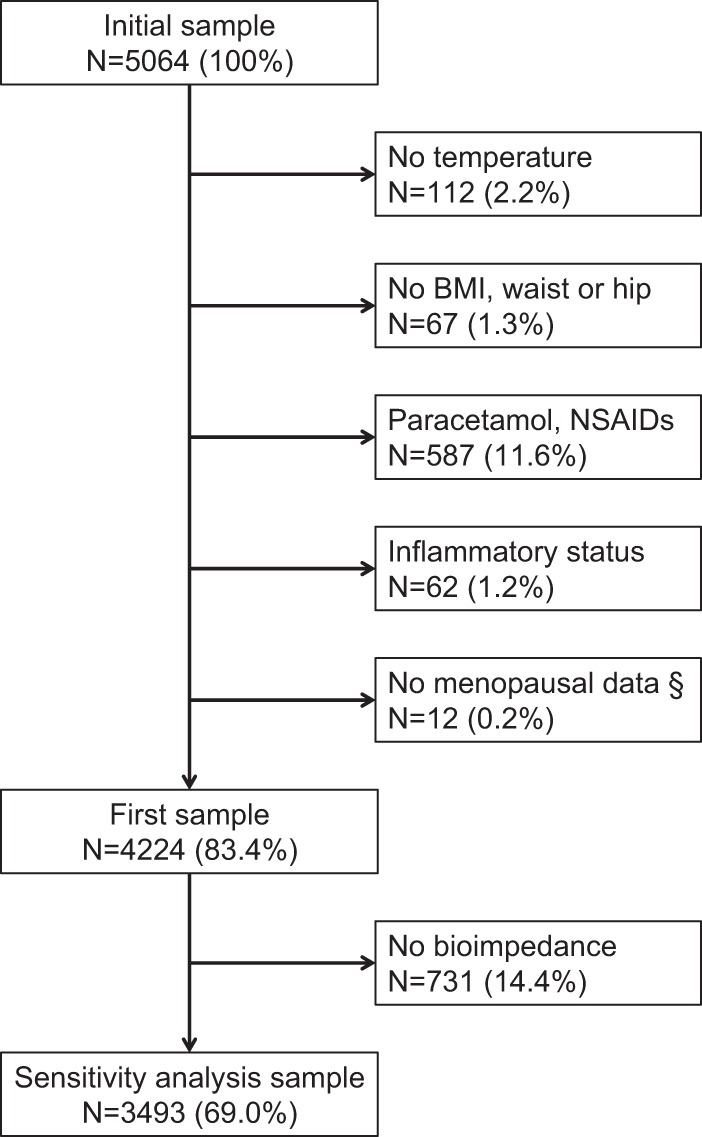
Selection procedure. BMI body mass index; NSAIDs non-steroidal anti-inflammatory drugs. § women only

The selection procedure is summarized in Fig. 1. Of the initial 5064 participants, 4224 (83.4% of the initial sample) were retained for the main analysis. A further 731 participants (14.4%) had no bioimpedance data, leaving 3493 participants (69% of the initial sample size) for sensitivity analysis.

The characteristics of the included and the excluded participants are summarized in Supplementary table 1. Excluded participants were older, had a higher body temperature, were more frequently women or presented with diabetes, had higher BMI, waist and hip levels and had higher hs-CRP and insulin levels. The characteristics of the sample according to gender and menopausal status are summarized in Table 1.

**Table 1:** Clinical characteristics of the participants, stratified by gender and menopausal status, CoLaus study, Lausanne, 2009–2012

	Men	Premenopausal women	Postmenopausal women
*N* (%)	2032 (48.1)	631 (14.9)	1561 (37.0)
Age (years)	56.8 ± 10.3	46.3 ± 3.6	62.3 ± 8.5
Temperature (°C)	36.1 ± 0.4	36.4 ± 0.4	36.3 ± 0.4
Body mass index (kg/m^2^)	26.9 ± 3.9	24.7 ± 4.7	25.5 ± 4.8
*BMI categories (%)*			
Underweight	8 (0.4)	16 (2.5)	40 (2.6)
Normal	668 (32.9)	379 (60.1)	772 (49.5)
Overweight	995 (49.0)	166 (26.3)	498 (31.9)
Obese	361 (17.8)	70 (11.1)	251 (16.1)
Waist (cm)	96.9 ± 11.2	83.9 ± 11.6	88.1 ± 12.6
Abdominal obesity (%)	593 (29.2)	202 (32.0)	746 (47.8)
Hip (cm)	100.8 ± 8.6	96.2 ± 10.9	99.4 ± 11.0
Waist to hip ratio	0.96 ± 0.06	0.87 ± 0.06	0.88 ± 0.06
Body area (m^2^)	2.00 ± 0.18	1.73 ± 0.17	1.73 ± 0.18
Resting heart rate (bpm)	66 ± 10	69 ± 9	68 ± 10
hs-CRP (mg/l)	2.1 ± 2.7	2.2 ± 2.8	2.3 ± 2.6
Glucose (mmol/l)	6.1 ± 1.3	5.4 ± 0.8	5.7 ± 0.9
Insulin (µU/ml)	9.7 ± 22.2	6.6 ± 6.4	7.8 ± 5.9
Diabetes (%)	306 (15.1)	10 (1.6)	118 (7.6)

Results are expressed as mean ± standard deviation or as percentage

*BMI* body mass index, *bpm* beats per minute, *hs-CRP* high-sensitivity C-reactive protein

### Association of body temperature with adiposity and metabolic markers

The bivariate associations of body temperature with adiposity and metabolic markers, stratified by gender and menopausal status, are summarized in Table 2. Body temperature was positively associated with WHR, resting heart rate and insulin in both genders. In men and postmenopausal women, body temperature was positively associated with BMI, waist, body area, and glucose level. Positive associations between body temperature and age, hip, and hs-CRP levels were also observed in men. In premenopausal women, body temperature was negatively associated with age.

**Table 2:** Bivariate associations of body temperature with adiposity and metabolic markers, stratified by gender and menopausal status, CoLaus study, Lausanne, 2009–2012

	Men	Premenopausal women	Postmenopausal women
Age (years)	0.059**	−0.102*	−0.023
Body mass index (kg/m^2^)	0.157***	0.055	0.083**
Waist (cm)	0.163***	0.071	0.104***
Hip (cm)	0.083***	0.016	0.050
Waist to hip ratio	0.187***	0.096*	0.132***
Body area (m^2^)	0.094***	0.031	0.085***
Resting heart rate (bpm)	0.227***	0.140***	0.182***
hs-CRP (mg/l)	0.111***	0.058	0.037
Glucose (mmol/l)	0.104***	0.065	0.088***
Insulin (µU/ml)	0.148***	0.170***	0.117***

Results are expressed as Spearman correlation coefficients: *p < 0.05; **p < 0.01; ***p < 0.001

*bpm* beats per minute, *hs-CRP* high-sensitivity C-reactive protein

The multivariable analysis of the associations of body temperature with adiposity markers, stratified by gender and menopausal status, are summarized in Table 3. The associations were adjusted for age, resting heart rate, hs-CRP, and insulin. In men and postmenopausal women, body temperature was positively associated with waist and WHR, and with BMI in men; no associations were found between body temperature and hip or body area. In premenopausal women, no associations were found between body temperature and all obesity markers studied (Table 3). The associations between heart rate and body temperature remained significant irrespective of the obesity marker considered; the association between insulin levels and body temperature remained significant in both genders, while the association between hs-CRP and body temperature was only significant in men (supplementary table 2).

**Table 3:** Multivariable-adjusted associations of body temperature with adiposity markers, stratified by gender and menopausal status, CoLaus study, Lausanne, 2009–2012

	Men	Premenopausal women	Postmenopausal women
Body mass index (kg/m^2^)	0.112***	−0.021	0.038
Waist (cm)	0.111***	−0.003	0.076*
Hip (cm)	0.024	−0.060	−0.004
Waist to hip ratio	0.133***	0.076	0.122***
Body area (m^2^)	0.057	−0.043	0.037

Results are expressed as standardized regression coefficients. Analysis by linear regression using body temperature as dependent variable and adjusting for age, resting heart rate, high-sensitivity C-reactive protein (log-transformed) and insulin (log-transformed): *p < 0.05; **p < 0.01; ***p < 0.001 a correlation with p < 0.001 is much stronger (***) than p < 0.01 (**) or p < 0.05 (*).

**Fig. 2:**
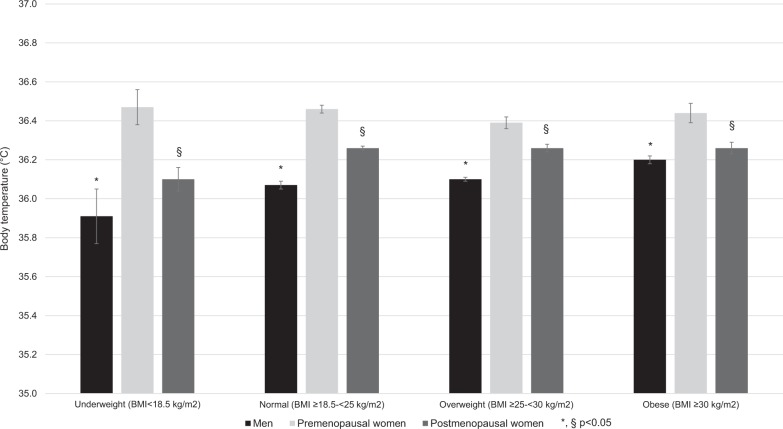
Association of body temperature with body mass index categories, stratified by gender and menopausal status, CoLaus study, Lausanne, 2009–2012

The bivariate and multivariable associations of body temperature with BMI categories are summarized in Fig. 2. In men and postmenopausal women, an increase in body temperature was found from underweight to obese participants after adjusting for age, resting heart rate, hs-CRP, and insulin. In premenopausal women, no differences in body temperature were found between BMI categories.

### Sensitivity analyses

The bivariate and multivariable associations of body temperature with body composition, stratified by gender, and menopausal status, are summarized in supplementary table 3. On bivariate analysis, body temperature increased with fat mass (% weight, kg, and kg/m^2^) in men and postmenopausal women. Similar associations were obtained after multivariable analysis adjusting for age, resting heart rate, hs-CRP and insulin in men, while the associations in postmenopausal women were no longer significant.

## Discussion

To our knowledge, this is the second largest study assessing the association between body temperature and obesity markers. Our results show that in men and in postmenopausal women, body temperature is positively associated with obesity markers, while in premenopausal women no significant association was found after multivariable adjustment.

### Body temperature and anthropometric markers

BMI, waist, hip, and WHR were positively associated with body temperature in men and postmenopausal women. A positive association between BMI and body temperature had already been reported in some studies [8–10], but not in others [11–13]. A possible explanation for the lack of association in the last studies is that they were conducted in small samples and had thus a reduced statistical power. The fact that body temperature was also positively associated with waist and WHR further suggests it is increased adiposity that leads to a higher body temperature. Indeed, in the sensitivity analyses, fat mass index (kg/m^2^) showed the strongest association with body temperature in men (Supplementary table 3).

As obese subjects have a larger body surface area, loss of temperature to the environment would be more important in obese. Still, on bivariate analysis, a positive association between body temperature and body surface area was found, but this association was no longer significant after multivariable adjustment. Overall, our results suggest that the increase body area of obese subjects does not influence significantly their temperature.

### Body temperature and metabolic markers

The chronotropic effect of temperature has been widely documented [20]. A study by Jose et al. identified a 7.15 ± 0.19 bpm increase per 1 °C elevation in internal temperature in humans [21]. Heart rate is also associated with obesity: autonomic regulation toward sympathetic activation with or without simultaneous parasympathetic inhibition in obese subjects compared to lean peers is described [22].

A strong association between glucose or insulin levels with body temperature was found. The association between body temperature and insulin persisted after adjustment for obesity markers in women and to a lesser degree in men, a finding also reported elsewhere [23]. Overall, our results suggest that insulin could exert a thermogenic effect independently of obesity levels, possibly by direct interaction with warm-sensitive neurons stimulating active brown adipose tissue (BAT) [23]. BAT activity was not assessed in this study. However, there is a known inverse relationship between BAT activity and adiposity, so it is unlikely that BAT activation would explain the higher temperature observed with obesity in this population.

No associations between body temperature and obesity or metabolic markers were found in premenopausal women. The most likely explanation is that menstrual cycle has a stronger effect than the markers studied. Indeed, in young women, fluctuations in body temperature between the luteal and follicular phases may be >0.5 °C, which would cancel out smaller variations due to other factors. Conversely, after menopause, body temperature decreases over the whole day [24], thus allowing the detection of smaller differences.

Thus, the higher temperature observed among obese subjects could be due to several mechanisms. First, obese subjects have a higher resting metabolic rate [9], a feature also observed in this study by the positive association between resting heart rate and body temperature. Adipose tissue is a complex, highly active endocrine organ, secreting hormones, such as leptin, adiponectin, and cytokines (adipokines) [25]. These hormones have a strong effect on thermogenesis and energy homeostasis: leptin has a thermogenic effect via increased heat production in skeletal muscle [26, 27], and many hypothalamic neurons involved in regulating non-shivering thermogenesis are also leptin sensitive [28]. Finally, large-scale alterations of the gut microbiota are associated with obesity and microbiota composition changes with weight loss [29]. Gut microbiota can affect host metabolism via signaling pathways in the gut, with effects on inflammation, insulin resistance and deposition of fat stores [30]. Accordingly, one could speculate that there could be an indirect relationship between intestinal microbiota composition and thermal homeostasis in humans, as recently described in mice [31].

### Body temperature and age

In contrast to the study of Waalen [10], we found a positive correlation between body temperature and age in men. The higher prevalence of obesity with age could explain this correlation. However, it is not excluded that very old men do have a lower body temperature; the population over 80 years of age was not included in our study. By contrast, the temperature decreases with the years in women. This observation can be reinforced with the beginning of menopause, with a lower body temperature linked to the disappearance of the menstrual cycle.

### Study strengths and limitations

This study was conducted in a general population, allowing the generalization of the results to similar populations of Caucasian descent. Its large sample size and the variety of data collected also allowed assessing the associations between body temperature and a range of obesity and metabolic markers.

This study also has some limitations. First, body temperature was assessed using a tympanic thermometer on a single occasion, while the gold standard for clinical thermometry is the pulmonary artery catheter thermometer [32]. Still, such measure would be unethical to perform in free-living, healthy subjects, and it has been shown that non-invasive, tympanic membrane measurement accurately assesses core body temperature compared to reference methods [33–35]. Second, no information about the follicular phase in women was documented; hence, the associations in premenopausal women were blunted as no adjustment for follicular phase was possible. Future studies on this topic should gather information regarding follicular phase to identify determinants of body temperature in this group. Third, no information regarding the thyroid hormone status was collected or about polycystic ovarian syndrome in women was documented. Finally, beta blocker treatment was not considered in the analysis; beta blockers have been shown to increase core temperature in animal models [36]. As beta blockers reduce heart rate, one may speculate that the effect in humans would be to decrease metabolism and body temperature [37].

## Conclusion

Body temperature is associated with obesity markers in men and postmenopausal women. The absence of association between body temperature and adiposity markers in premenopausal women might be due to the menstrual cycle.

## Electronic supplementary material

Supplementary tables
