# Supplementary material for: Association of body temperature with obesity. The CoLaus study
Source: Int J Obes (Lond). 2018 Sep 24;43(5):1026–33. doi: 10.1038/s41366-018-0218-7 (PMC6760582; doi:10.1038/s41366-018-0218-7)
Supplement: Supplementary file 1 — Supplementary tables [file 41366_2018_218_MOESM1_ESM.docx]

### Supplementary tables

**Supplementary table 1**. Clinical characteristics of the included and excluded participants, CoLaus study, Lausanne, 2009-2012.

|  | Included | Excluded | P-value |
| --- | --- | --- | --- |
| N (%) | 4224 (83.4) | 840 (16.6) | - |
| Women (%) | 2192 (51.9) | 515 (61.3) | <0.001 |
| Age (years) | 57.3 ± 10.4 | 60.2 ± 11.0 | <0.001 |
| Temperature (°C) | 36.2 ± 0.4 | 36.3 ± 0.4 | <0.001 |
| Body mass index (kg/m^2^) | 26.0 ± 4.5 | 27.1 ± 5.2 | <0.001 |
| Waist (cm) | 91.7 ± 12.9 | 93.6 ± 13.5 | <0.001 |
| Hip (cm) | 99.6 ± 10.0 | 102.2 ± 10.9 | <0.001 |
| Waist to hip ratio | 0.92 ± 0.07 | 0.91 ± 0.08 | 0.102 |
| Body surface area (m^2^) | 1.86 ± 0.22 | 1.86 ± 0.22 | 0.934 |
| Resting heart rate (beats.min^-1^) | 67.3 ± 9.7 | 68.8 ± 9.9 | <0.001 |
| Hs-CRP (mg/l) § | 2.2 ± 2.7 | 4.5 ± 6.3 | <0.001 |
| Glucose (mmol/l) | 5.9 ± 1.2 | 5.9 ± 1.3 | 0.944 |
| Insulin (µu/ml) § | 8.5 ± 16.0 | 9.8 ± 14.8 | <0.001 |
| Diabetes (%) | 434 (10.3) | 105 (12.8) | 0.033 |

Hs-CRP, high sensitivity C-reactive protein. Results are expressed as mean ± standard deviation or as percentage. Between-group comparisons performed using chi-square for categorical variables and student’s t-test or Kruskal-Wallis test (§) for continuous variables.

**Supplementary table 2.** Bivariate associations of core body temperature with adiposity and metabolic markers, only participants with all adiposity markers, stratified by gender and menopausal status, CoLaus study, Lausanne, 2009-2012.

|  | Men | Premenopausal women | Menopausal women |
| --- | --- | --- | --- |
| N (%) | 1610 (49.7) | 485 (15.9) | 1147 (35.4) |
| Age (years) | 0.051 * | -0.083 | -0.025 |
| Body mass index (kg/m^2^) | 0.166 *** | 0.076 | 0.088 ** |
| Waist (cm) | 0.170 *** | 0.088 | 0.090 ** |
| Hip (cm) | 0.087 *** | 0.038 | 0.046 |
| Waist to hip ratio | 0.191 *** | 0.104 * | 0.113 *** |
| Body area (m^2^) | 0.102 *** | 0.055 | 0.073 * |
| Fat mass (% weight) | 0.187 *** | 0.074 | 0.083 ** |
| Fat mass (kg) | 0.184 *** | 0.076 | 0.096 ** |
| Fat mass index (kg/m^2^) | 0.198 *** | 0.075 | 0.098 *** |
| Non-fat mass (kg) | 0.042 | 0.019 | 0.032 |
| Non-fat mass index (kg/m^2^) | 0.090 *** | 0.058 | 0.045 |
| Heart rate (beats per min) | 0.240 *** | 0.168 *** | 0.183 *** |
| Hs-CRP (mg/l) | 0.136 *** | 0.088 | 0.044 |
| Glucose (mmol/l) | 0.109 *** | 0.077 | 0.091 ** |
| Insulin (µU/ml) | 0.150 *** | 0.187 *** | 0.137 *** |

Hs-CRP, high sensitivity C-reactive protein. Results are expressed as Spearman correlation coefficients: *, p<0.05 ; **, p<0.01 ; ***, p<0.001.

**Supplementary table 3.** Multivariate associations of core body temperature with adiposity markers, only participants with all adiposity markers, stratified by gender and menopausal status, CoLaus study, Lausanne, 2009-2012.

|  | Men | Premenopausal women | Menopausal women |
| --- | --- | --- | --- |
| Body mass index (kg/m^2^) | 0.121 *** | -0.003 | 0.036 |
| Waist (cm) | 0.108 *** | -0.001 | 0.041 |
| Hip (cm) | 0.025 | -0.049 | -0.021 |
| Waist to hip ratio | 0.128 *** | 0.062 | 0.084 ** |
| Body surface area (m^2^) | 0.058 * | -0.027 | 0.014 |
| Fat mass (% weight) | 0.137 *** | -0.015 | 0.068 * |
| Fat mass (kg) | 0.130 *** | -0.016 | 0.050 |
| Fat mass index (kg/m^2^) | 0.145 *** | 0.003 | 0.056 |
| Non-fat mass (kg) | 0.019 | -0.021 | -0.023 |
| Non-fat mass index (kg/m^2^) | 0.053 | -0.008 | -0.016 |

§, analysis performed on log-transformed data. Results are expressed as standardized regression coefficients. Analysis by linear regression using core body temperature as dependent variable and adjusting for age, resting heart rate, high sensitivity C-reactive protein (log-transformed) and insulin (log-transformed): *, p<0.05 ; **, p<0.01 ; ***, p<0.001.
